# Solitary plasmacytoma of the tibia: literature review and case report

**DOI:** 10.3389/fonc.2026.1503479

**Published:** 2026-01-21

**Authors:** Xianwen Hu, Dandan Li, Xiao Hu, Shun Li, Pan Wang

**Affiliations:** 1 Department of Nuclear Medicine, Affiliated Hospital of Zunyi Medical University, Zunyi, China; 2 Department of Obstetrics, Zunyi Hospital of Traditional Chinese Medicine, Zunyi, China

**Keywords:** MRI, plasma cell myeloma, solitary plasmacytoma, SPECT, tibia

## Abstract

Solitary plasmacytoma (SP) is seldom encountered. It can affect any bone in the body, but is more common in the spine, especially in the thoracic region, while SP in the tibia is relatively rare. Herein, we report the case of a 34-year-old woman who visited our hospital with right calf pain for over a month. The X-ray, computed tomography (CT), and magnetic resonance imaging (MRI) revealed a tumor growing along the longitudinal axis of her right tibia, which was suspected to be malignant. The patient subsequently underwent a puncture biopsy, and the pathological results revealed a plasma cell myeloma. To further evaluate the extent of tumor involvement, the patient underwent single-phase technetium-99 labeled methylene diphosphonate (^99m^Tc-MDP) single photon emission computed tomography (SPECT) whole-body bone imaging, and the results showed no significant radioactive concentration in the entire body except for the right proximal tibia. Based on these imaging features and pathological results, the patient was diagnosed with SP. We summarized the clinical features and imaging findings of tibial SP based on our case and the published literature The results showed that SP is more likely to occur in young people. Its imaging has a certain specificity, which is characterized by a uniform low - density shadow growing along the longitudinal axis, without a sclerotic rim, and increased radioactive uptake on whole - body bone imaging. MRI showed long signals on T1 and T2, with significant enhancement on contrast-enhanced scans, but rarely breaking through the bone cortex to form soft tissue masses. The current study suggests that a thorough understanding of the clinical and imaging characteristics of the tibial SP can increase the likelihood of obtaining an accurate diagnosis before surgery.

## 1 Introduction

Plasmacytomas are clonal plasma cell proliferative neoplasms and are the second most malignant tumors of the hematologic system after leukemia (1). Depending on the extent of involvement of the lesion, they are categorized as either solitary plasmacytoma (SP) and multiple myeloma (MM) (2). SP is clinically rare, with an incidence of less than 5% of MMs (3). According to the site of lesion involvement, SP can be divided into SP of bone (SPB) and extramedullary plasmacytoma, and SPB can affect any bone in the body, but it is more common in the spine, especially the thoracic vertebra (4). SP in the tibia is rare; to our knowledge, only eight cases have been published to date. Imaging diagnostic methods for SP include traditional X-ray, computed tomography (CT), and magnetic resonance imaging (MRI)computed tomography (CT), single photon emission computed tomography (SPECT). Furthermore, positron emission tomography (PET)/CT is also of crucial importance in the diagnosis and management of SP, especially in evaluating for progression and other extramedullary organ or tissue involvement after treatment (5). Here, we report a pathologically proven SP of the tibia after surgical resection, describe its radiographic features and review the relevant published literature. The aim is to improve clinicians’ understanding of this rare disease.

## 2 Case description

A 34-year-old woman with right calf pain for over a month visited our hospital for medical help on December 17th, 2018. Physical examination showed obvious tenderness in the upper right leg of the patient; no obvious mass was felt, and no obvious positive signs were found in other body parts. The patient denied that she and her family had any history of genetic diseases, tumors, or other significant medical conditions, including hepatitis and tuberculosis.

**Figure 1:**
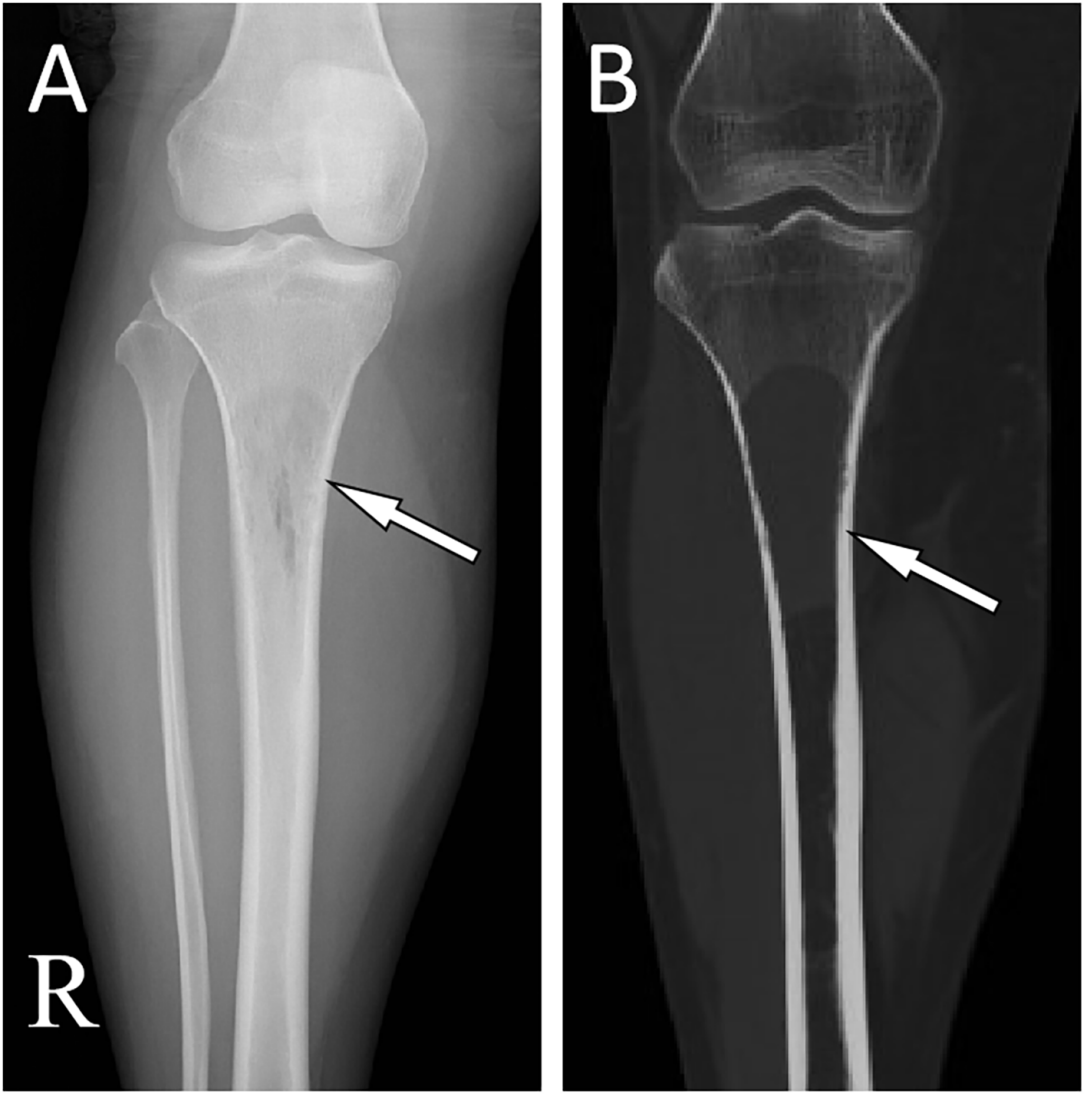
**(A)** X-ray shows a low-density shadow along the longitudinal axis at the proximal end of the right tibia without periosteal reaction and sclerotic rims (arrow). **(B)** CT showed that the normal trabecular structure of the proximal end of tibia disappeared, the cortical bone was intact, and no obvious soft tissue mass was found (arrow).

**Figure 2:**
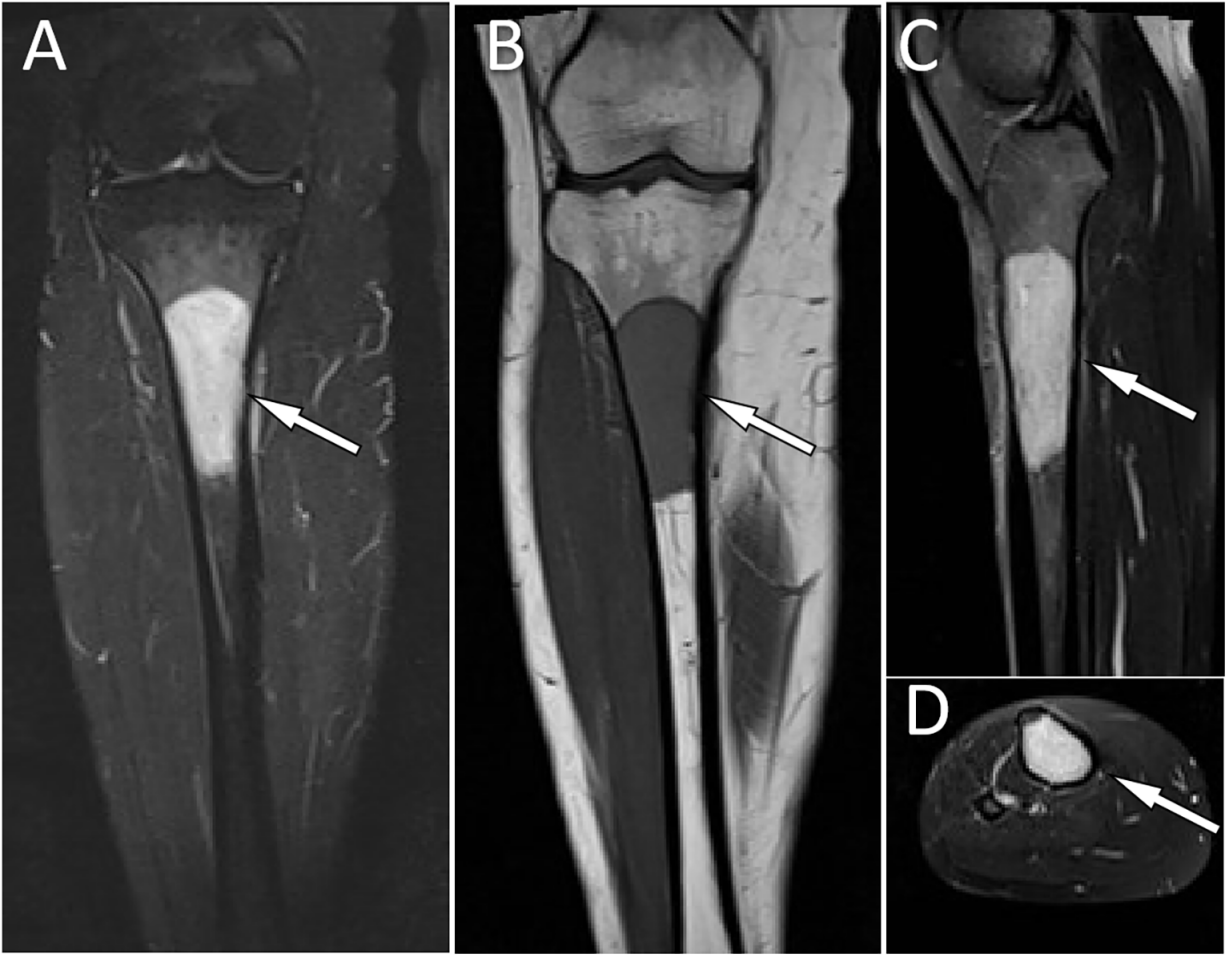
MRI showed a homogeneous signal of the lesion, with hypersignal on T2WI **(A)** and hyposignal on T1WI **(B)**. Contrast-enhanced T1WI showed significant homogeneous enhancement **(C)**, coronal and **(D)**, axial.

**Figure 3:**
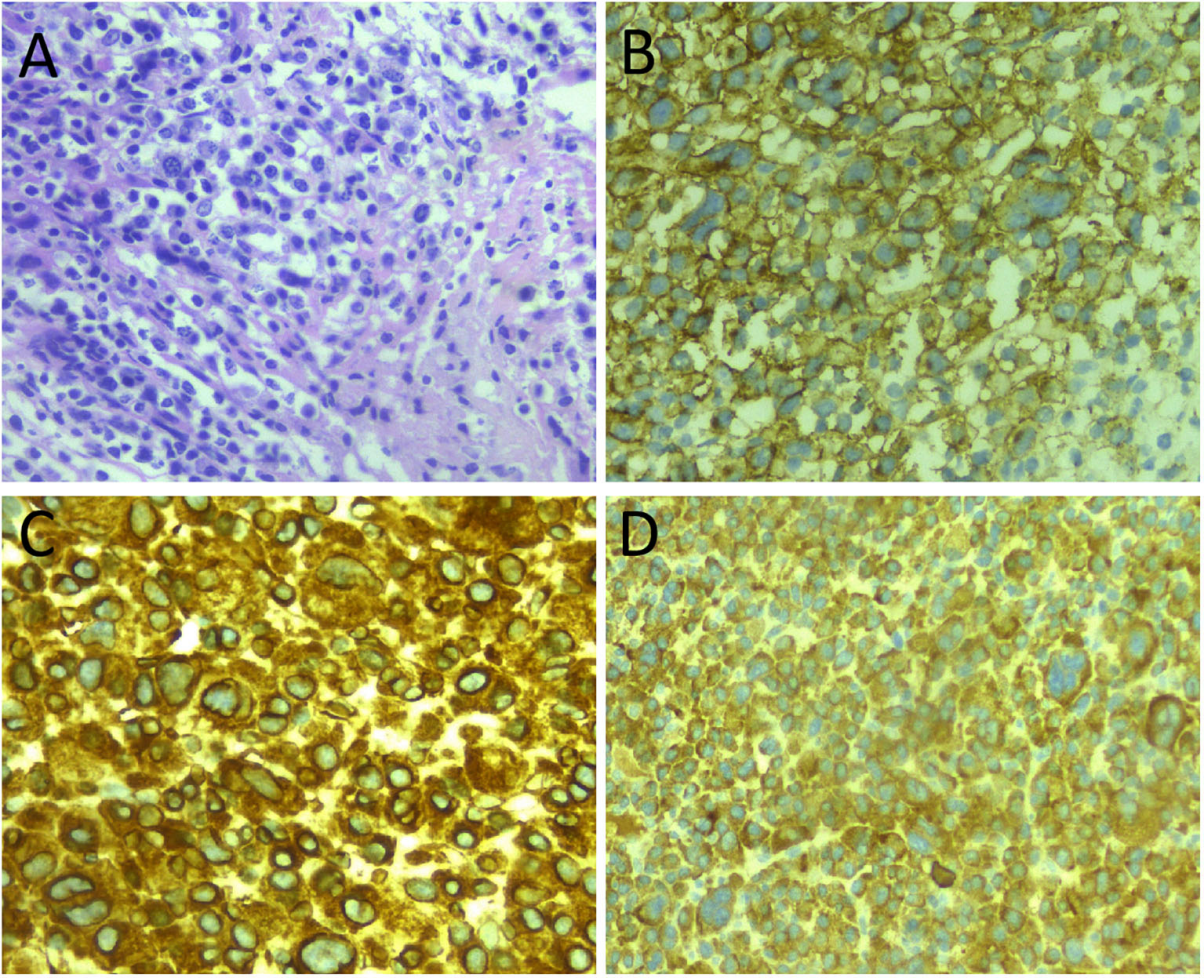
**(A)** Hematoxylin and eosin (H&E) staining showed diffusely distributed tumor cells with round nuclei and abundant cytoplasm. Immunohistochemistry showed positive expression of tumor cells CD138 **(B)**, Kappa **(C)**, and MUM1 **(D)**. All images are 200× magnification.

**Figure 4:**
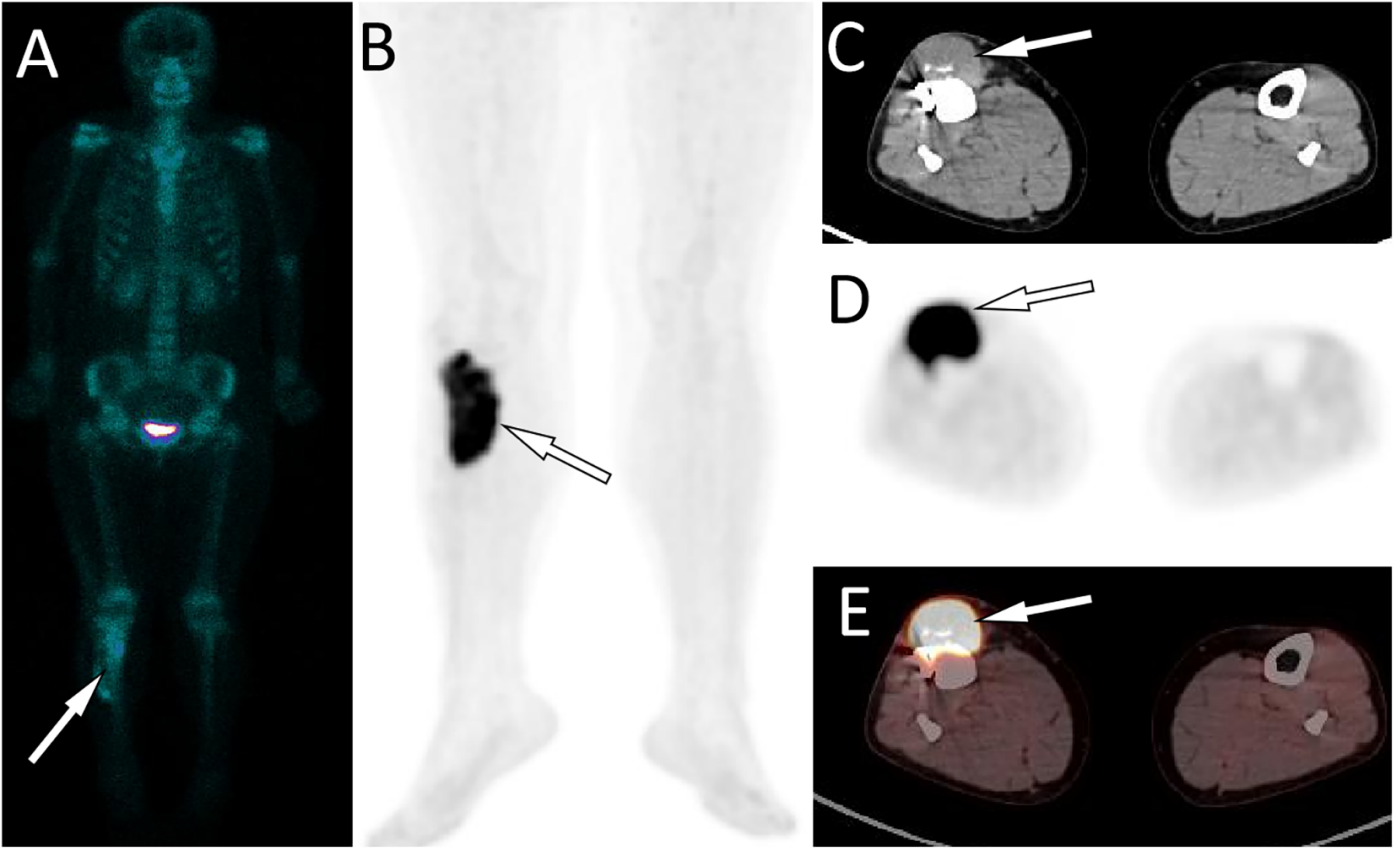
^99m^Tc-MDP SPECT whole body bone imaging showed no significant radioactive concentration except for the right proximal tibia (**A**, arrow). The PET/CT examination of the patient about 2 years after surgery revealed an increased ^18^F-FDG uptake in the surgical area of the original proximal tibial lesion (**B**, arrow). The axial CT **(C)** at the corresponding position reveals a soft - tissue mass shadow of uniform density at the anterior edge of the original proximal tibial surgical area (arrow), measuring approximately 4.0 cm × 1.8 cm × 8.5 cm in size. It is accompanied by a significantly increasded ^18^F - FDG uptake on the axial PET (**D**, arrow) and PET/CT fusion (**E**, arrow), with a SUVmax of 14.1.

Laboratory tests, including blood routine, liver function, renal function and serum tumor markers, were all within the normal reference value range. Plain X-ray and CT revealed a low-density bone destruction in the patient’s right upper tibia (as shown in **Figure 1**), which showed hyposignal on T1-weighted imaging (T1WI) and hypersignal on T2-weighted imaging (T2WI) on MRI, with obvious enhancement on contrast-T1WI (as shown in **Figure 2**). These imaging findings are suggestive of a neoplastic lesion. The patient underwent an open biopsy of the right tibial lesion on December 21, 2018, to further determine the treatment plan. Pathological findings (as shown in **Figure 3**) showed that the tumor cells were round and oval, resembling mature plasma cells, with positive expressions of CD138, MUM1, CD79a, Kappa, CD56 were positive, and proliferation index by Ki67 was 20%, but the cells did not express CD20, CD3, Lambda, LCA, PAX-5. Based on these pathological features and immunohistochemical findings the patient was diagnosed with plasma cell myeloma. Subsequently, she underwent technetium-99 labeled methylene diphosphate (^99m^Tc-MDP) SPECT whole-body bone imaging, which showed no skeletal abnormalities except for increased radioactivity uptake in the right tibia (**Figure 4A**). Moreover, she also underwent an iliac bone puncture and the bone marrow examination results did not show any clonal plasma cells, nor was there any infiltration of malignant tumor cells. The results of serum immunoglobulin and urine Bence-Jones protein tests were both negative, and k/λ ratio is 1.48. Based on the above imaging findings, laboratory tests, pathological and immunohistochemical results of the patient, the patient was diagnosed with SP of the tibia. After communicating with the patient, she opted for surgical resection of right tibial lesion, argon knife inactivation and bone cement filling on December 28, 2018. Postoperatively, the patient was discharged from the hospital on January 5, 2019, after treatment including pain relief, hemostasis, prevention of thrombosis and prevention of infection.

The patient returned to the hospital on November 4, 2021, with right calf pain for over a month. On physical examination, a mass was palpable on the medial side of the right calf, measuring about 2.0cm×1.2cm, with poor mobility, obvious pressure pain, and no other obvious positive signs. The patient subsequently underwent X-ray and MRI examinations. No significant abnormalities were found on X-ray, while an abnormal signal shadow around the operative area of the upper leg was observed on MRI. To further evaluate the patient’s condition, the patient subsequently underwent a positron emission tomography (PET)/CT scan, which showed intense fluorin-18 labeled fluorodeoxyglucose (^18^F-FDG) uptake in the soft tissue mass in the surgical area, with no abnormally elevated radioactivity uptake throughout the rest of the body (as shown in **Figures 4B–E**). Based on these imaging findings, the patient was suspected of having a local tumor recurrence. As the lesion was limited, she was referred to the Department of Radiation Therapy on November 18, 2021, for radiation therapy at 50 Gy, approximately 2.0 Gy per day. The patient was followed up regularly by telephone every three months.

On January 28, 2025, the patient came to the hospital again for medical assistance after discovering a soft tissue mass with poor mobility at the site of her right calf surgery. Lower limb CT examination suggested the possibility of tumor recurrence. Subsequently, she began to receive radiotherapy of 50 Gy, also at approximately 2.0 Gy per day. The most recent CT scan was performed on March 31, 2025, and the results indicated that the volume of the mass had decreased compared to before. We suggested that the patient undergo whole body PET/CT scan to further assess whether the condition had progressed, but the patient refused due to the inability to afford the cost of the examination. The patient is still under follow-up observation at present.

## 3 Literature review

Case reports or series of SP of the tibia published in English up to August 1, 2023, were searched from PubMed, Embase, and Web of Science databases. The retrieval formula was (tibia*) AND (solitary plasmacytoma). After searching by keywords and carefully reading the original articles, eight articles containing information on eight patients were finally included (6–13). The clinical features of nine cases of SP, including the patient we reported and the imaging manifestations, are summarized in **Table 1**. Of the nine patients, including five males and four females, the age range at diagnosis was 14 to 58 years, with a median age of 29. Most lesions (7/9) were located in the proximal part of the tibia, and all had clinical symptoms of pain at the lesion lasting for varying periods. Radiographic and CT signs were described in 8 out of 9 patients, presenting as hypodense or cystic densities with longitudinal growth without a sclerotic rim. A few cases described MRI and SPECT findings; on MRI, the lesions all showed low signal on T1WI and high signal on T2WI. Increased radioactive uptake was demonstrated on ^99m^Tc-MDP SPECT.

**Table 1:** The clinical and imaging features of patients with solitary plasmacytoma of the tibia.

Case	Author/year/country	Gender/age	MS/DT(months)	Location	X-ray/CT				MRI		^99m^Tc-MDP SPECT	Management	Follow-up (months)
					Density	bone cortex	Growth pattern	sclerotic rim	T1WI	T2WI			
1 (5)	Rago/2010/Italy	M/22	Pain/1	R/far-end	low	integrity	longitudinal axis	no	–	–	–	Radiotherapy	–
2 (6)	Madi/2018/India	M/58	Pain/6	R/near-end	low	integrity	longitudinal axis	no	hypo-	hyper-	–	Surgery+Radiotherapy	–
3 (7)	Yang/2023/China	M/47	Pain/osteomyelitis 32 years	R/near-end	low	integrity	longitudinal axis	no	hypo-	hyper-	strong radioactive uptake	Surgery+Radiotherapy	12/AWD
4 (8)	Yamaç/2002/Turkey	F/29	Pain/2	R/near-end	low	integrity	longitudinal axis	no	–	–	–	–	–
5 (9)	Kumar/2011/India	F/14	Pain/12	L/near-end	low	integrity	longitudinal axis	no	–	hyper-	–	Radiotherapy	34/AWD
6 (10)	Bertoni/1998/Brazil	F/17	pain, swelling/36	R/far-end	low	integrity	longitudinal axis	no	–	–	strong radioactive uptake	Radiotherapy	8/AWD
7 (11)	Ishida/1995/USA	M/21	Lump, pain/12	R/near-end	low	integrity	longitudinal axis	no	hypo-	hyper-	strong radioactive uptake	–	–
8 (12)	Herranz/2000/Spain	M/45	–	R/-	–	–	–	–	–	–	–	Radiotherapy	22/Progression to multiple myeloma
9	Present case	F/34	Pain/1	R/near-end	low	integrity	longitudinal axis	no	hypo-	hyper-	strong radioactive uptake	Surgery+Radiotherapy	18/AWD

M, male; F, female; L, left; R, right; AWD, alive without disease; MD, Maximum diameter; ERG, erythroblast specific transformation related genes; MS, Main symptoms; DT, duration time; MRI, magnetic resonance imaging; T1WI, T1-weighted imaging; T2WI, T2-weighted imaging; CT, computed tomography; ^99m^Tc-MDP, technetium-99 labeled methylene diphosphate; SPECT, Single photon emission computed tomography; hypo-, hyposignal; hyper-, hypersignal.

## 4 Discussion

SP is a separate subtype of plasma cell myeloma characterized by localized abnormal proliferation of neoplastic monoclonal plasma cells and is classified into SP of bone (SPB) and extramedullary plasmacytomas, according to the site of involvement of the lesion (14). SPB accounts for 70% of all isolated plasmacytomas, is twice as common in men as in women. Its median age of onset is at 55, ten years earlier than the onset of multiple myeloma (4). Our study showed that the incidence of SP in the tibia did not differ significantly between men and women and that the onset was younger, with a median age of 29 years. It occurs predominantly in the spine and rarely in the tibia, and the results of our systematic search showed that there were only eight cases published in English before us. Pain is the main clinical manifestation of this disease, with localized soft tissue swelling or lumps present in a minority of patients (11, 12). It is worth mentioning that there is a case in the literature with SP of the tibia who had a history of chronic osteomyelitis for up to 32 years prior (8), suggesting that this may be a risk factor for the development of it, but this needs to be confirmed with more cases in future studies.

Imaging studies, including plain radiographs, CT and MRI, are necessary for proper preoperative diagnosis of bone lesions and treatment planning. Our results from a systematic literature review showed that SP favors the right tibia and is prevalent in the proximal tibia. On radiographs or CT, tibial SP shows a hypodense or cystic density shadow that grows along the longitudinal axis, with a well-defined, homogeneous density, no sclerotic rim, and the lesion rarely breaks through the bone cortex to invade the surrounding soft tissues (6–12). On MRI, the lesion showed a homogeneous low signal at T1WI and a high signal at T2WI (7, 8, 10, 12). The radiographic and CT findings of this patient are consistent with those reported in the literature above. Besides, our patient underwent a contrast-enhanced T1WI scan and showed significant homogeneous enhancement, suggesting a rich blood supply to the lesion. Moreover, nuclear medicine molecular imaging, including SPECT whole body bone imaging and PET/CT imaging, is also necessary for patients after the histopathology of plasmacytoma has been confirmed, as it can show the presence of lesions at other locations in addition to the local lesions shown on conventional plain radiographs, CT and MRI to rule out the possibility of multiple myeloma. As in the patient we reported, on whole-body bone imaging, all lesions of tibial SP showed strong ^99m^Tc-MDP uptake, whereas the rest of the skeleton did not (8, 11, 12). The imaging differential diagnosis of SP in the tibia includes other osteolytic osteoid bone-destroying lesions such as bone cysts, aneurysmal bone cysts (ABC), giant cell tumor of bone (GCTB), and osteofibrous dysplasia. Bone cysts are most common in children under 20 years of age and occur before epiphyseal healing, and they also develop along the longitudinal axis of the diaphysis. However, on contrast-enhanced T1WI, bone cysts have only marginal enhancement. In contrast, SPs have marked enhancement at the margins and within the lesion, which is not difficult to distinguish from other bone cysts (15). ABCs are eccentric, hypodense osteolytic and mildly distensible changes, with thinning of the bone cortex in the form of a shell, which a sclerotic rim of bone may surround, and in some lesions, there is a localized interruption of the bone cortex and formation of a soft-tissue mass towards the outside of the bone (16). On MRI, T1WI showed that its signal was equal to or slightly lower than that of the muscle; T2WI showed that the lesion was of inhomogeneous high signal, and the fibers and septum, bone spurs, and iron-containing protein deposits within the lesion were of hyposignal, and a small fluid surface could be seen in the larger lesion, and the lesion was markedly inhomogeneous in enhancement on contrast-enhanced scans (17). GCTB usually occurs at the end of metaphyseal healing in the extremities and is usually characterized by expansive and eccentric bone destruction; the bone shell is thin, and the contour is usually intact, in which a slender bone ridge is seen, constituting a compartmentalized shape; the border between the damaged bone and normal bone is clear but not sharp, without sclerotic rim, and areas of hypodense necrosis are seen within the tumor; and MRI showed low or moderate signal on T1WI and mixed-signal on T2WI (18). Osteofibrous dysplasia can appear three kinds of changes on X-ray and CT: ground glass-like, cystic dilatation and loofah; in cystic dilatation changes, there is a sclerotic rim around the lesion, hyposignal on T1WI and bright hypersignal on T2WI of MRI, with clear boundaries, low signal linear segregation shadow can be seen in the area, and the edge of the lesion shows linear ring enhancement on enhancement, which is different from the obvious homogeneous enhancement of SP (19).

The diagnosis of SP was based on the diagnostic criteria of NCCN, which was mainly based on the histopathological evidence of monoclonal plasma cell proliferation and normal bone marrow appearance, combined with clinical evidence of no terminal organ damage and imaging evidence of no distant bone involvement and no multiple lesions, and MM and other diseases were excluded (20). Histologically, tumor tissue consists of neoplastic plasma-like cells. The poorly differentiated tumor cells had obvious atypia, distinct nucleoli and frequent nuclear schizophrenia. The highly differentiated tumor cells resembled normal plasma cells, with round and dislocated nuclei, abundant cytoplasm, basophilic or dichrophilic, and Russel’s bodies in the cytoplasm and Dutcher’s bodies in the nucleus can be seen in some tumor tissues (14, 21). The tumor cells of isolated plasmacytoma are light chain restricted; the tumor cells only express one light chain protein Kappa or Lambda. In addition, tumor cells often express plasma cell antibodies such as MUM1, CD38 and CDl38, and B cell labeled antibodies such as BOB1, OCT2 and CD79a are expressed to varying degrees, but CD20 is not expressed (22). The histology of the patient we reported showed diffusely distributed tumor cells with round nuclei and abundant cytoplasm; the tumor cells positively expressed Kappa, CD138, MUM1, CD79a, and CD56, but not CD20, CD3, Lambda, LCA, PAX-5, which was consistent with the diagnosis of plasmacytoma.

The main treatments for SP include surgery, radiotherapy, chemotherapy, and combination therapy (23). As SP lesions are more limited, most patients can choose surgery to remove the mass for treatment, which can improve the overall survival (OS) rate and cancer-specific survival of patients (14, 24), Plasmacytoma is sensitive to radiotherapy, so it can also improve the OS rate of plasmacytoma patients, and the overall response and complete response (CR) of radiotherapy to SP can reach over 90% and 50%, respectively (25). Adjuvant chemotherapy may be an option if relapse or risk of conversion to MM occurs after treatment (23). However, a previous study indicated that systemic therapy, whether used alone or in combination with RT, did not result in higher CR rates or longer survival time compared to radiotherapy alone (26). The prognosis of SP was better than that of MM, with a 2-year OS rate of 87.5% and 5- and 10-year OS rates of 78% and 54%, respectively (27, 28). Overall, the prognosis of SP remains poor. A recent study involving 175 patients showed that 40% of the patients experienced recurrence and 80% progressed to multiple myeloma during long-term follow-up, and the median progression-free survival was only 30 months (26). Our patient recurred two years after the first surgical removal of the mass and improved with radiation therapy. However, the patient we reported relapsed more than three years after the first radiotherapy when the condition was in remission, further revealing the nature of the disease being prone to recurrence.

Overall, SP occurring in the tibia is rare and more likely to occur in younger people. The imaging findings of SP of the tibia showed a uniform low-density shadow growing along the longitudinal axis without a sclerotic rim, which showed significant radioactive concentration on the SPECT whole-body bone imaging. On MRI, the lesions showed long T1 and T2 signals, significantly enhanced by contrast-enhanced scan but rarely broke through the bone cortex to form soft tissue masses.

## Data Availability

The original contributions presented in the study are included in the article/supplementary material. Further inquiries can be directed to the corresponding authors.
